# Classic literature and textbooks in perfusion, how they continue to influence

**DOI:** 10.1051/ject/2023014

**Published:** 2023-06-28

**Authors:** Joseph J. Sistino

When I was a student in 1974 there were two textbooks that had a major influence on my perfusion career. The first was Heart-Lung Bypass; Principles in Extracorporeal Circulation by Galletti and Brecher [1], and the other was Cardiac Surgery by John Norman [2]. At the time, both of these books were comprehensive reviews of the state-of-the-art of perfusion technology.

In Cardiac Surgery by John Norman, there was a chapter on the role of the perfusionist and the importance of academic perfusion education by Vasko and Dearing [3]. Jim Dearing emphasized perfusion as an emerging profession, as he was involved in the relatively new Ohio State perfusion education program. My own interest in university-based perfusion education had its beginnings in this chapter, and later I advocated for graduate level perfusion education in an editorial in this journal [4].

Heart-Lung Bypass was a very comprehensive review of perfusion research in the 1950s and the early 1960s. One area of interest that was stimulated by reading this book was blood conservation. I had read about the demands for blood products as cardiac surgery expanded in the early 1970’s, but didn’t realize that all available blood products would be needed for cardiac surgery unless something was done to reduce blood usage. Before there was hemodilution and before there was, cell washing devices, there was a technique used called “paired perfusion”.

This technique was discussed in Galetti’s book [5]. I had a hard time finding this publication when I was looking for references on blood conservation. I remember reading about double pump runs in Galetti’s book, and eventually found the reference. Since it was an old journal, I asked one of cardiac surgeons that I was working with if he had old Thoracic and Cardiovascular Surgery journals. I went to his office, and I found a bound copy of the journal from 1967. I was so excited, he gave me the bound copy, which I still have today.

The article on “paired perfusion” outlined the technique using the same heart-lung machine for two patients in a row. When I taught perfusion research class, this was usually the first paper that we reviewed because I wanted students to appreciate the history of cardiopulmonary bypass and vast amount of perfusion literature that’s available not just online, but also in libraries.

The research study was conducted at Boston Children’s Hospital. It was a very different time, when pediatric patients are averaged around 30 kg. The heart lung machine required 5 units of fresh heparinized blood to prime, and the disc oxygenators needed a whole day to be cleaned and sterilized between operations. This limited the amount of surgery that could be done. To reduce blood usage and allow more operations to be completed, the technique of using the same heart-lung-machine for two patients in a row was evaluated. I recently discovered that the lead author, a junior resident Robert Replogle, was responsible for cleaning the disc oxygenators.

Of course, the patients had to have the same blood type. The pump was primed with donor blood for the first patient, and the pump it was kept primed after the first operation. After the first operation the pump was wheeled into another room, for the second operation. At that point it contained mixture of donor blood and the first patient’s blood. This procedure was done in 197 patients. In one case the pump was contaminated and was not reused on a second patient.

One of the keys this success of this technique was the use of a rotating disc oxygenator. The disc oxygenator had a very low hemolysis rate, therefore the plasma hemoglobin levels at the end of the second operation were relatively low. The second patient benefited from the additional clotting factors from the first patient and had higher platelet and fibrinogen levels and less chest tube bleeding amounts, although there was no formal statistical analysis. The purpose of this paper, according to the title, was to show that was safe to use the heart-lung machine for an extended period of time. But I think the real purpose of this study was to reduce blood usage and increase the number of operations.

One can imagine if today someone submitted a research proposal to an IRB to do a similar project, how that would be quickly turned down. There are many instances of perfusion techniques that have been used in the past that have been rediscovered [6]. I’m not advocating for this particular technique since testing of blood products is now required, but using your imagination to develop research projects is the key to solving clinical problems. Only by being aware of the perfusion literature can you take advantage of previous knowledge and apply them in situations that arise in clinical situations that are new and challenging. As we celebrate the 70th anniversary of the first successful open heart operation by John H. Gibbon on May 7, 1953, I hope that you can appreciate the importance of perfusion history, and how so many things that we take for granted in cardiac surgery were carefully developed to solve these clinical challenges.

Joseph J. Sistino (PhD, CCP) Emeritus
